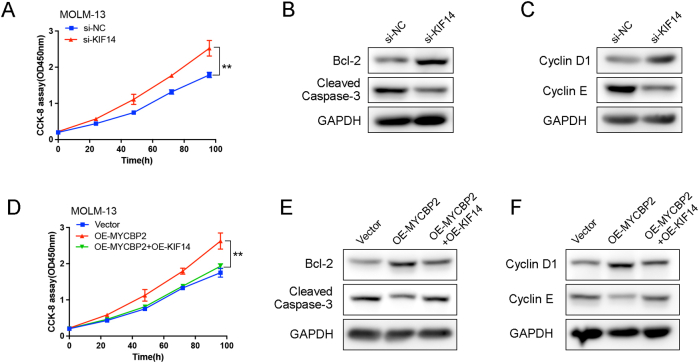# Ubiquitin E3 ligase MYCBP2 targets KIF14 and contributes to acute myeloid leukemia progression

**DOI:** 10.1016/j.jbc.2026.112204

**Published:** 2026-04-24

**Authors:** Guoli Yao, Yang Yang, Chunmei Chen, Bingrong Zheng, Tao Qiu, Lin Yang, Meiwei Hu

**Affiliations:** 1 Department of Hematology, First People's Hospital of Linping District, Hangzhou, China; 2 Department of Hematology, The Second Affiliated Hospital of Zhejiang Chinese Medical University, Hangzhou, China

**Keywords:** AML, MYCBP2, KIF14, ubiquitin-proteasome system

## Abstract

Acute myeloid leukemia (AML) is a challenging hematological malignancy characterized by poor clinical outcomes. This study investigates the role of MYCBP2 in AML progression, focusing on its interaction with KIF14 and its involvement in ubiquitin-mediated processes. We found that MYCBP2 is overexpressed in AML samples compared to normal tissues, with high expression correlating with adverse clinical outcomes. Knockdown of MYCBP2 using siRNA significantly inhibited cell proliferation and promoted apoptosis in MOLM-13 and HL-60 AML cell lines. Flow cytometry revealed that MYCBP2 knockdown leads to cell cycle arrest in the G0/G1 phase. Gene Set Enrichment Analysis indicated that MYCBP2 is associated with the Ubiquitin-Mediated Proteolysis pathway, regulating KIF14 protein stability through ubiquitination. High KIF14 expression was linked to better overall survival, and KIF14 knockdown partially reversed the effects of MYCBP2 knockdown on cell viability and apoptosis. *In vivo* studies demonstrated that MYCBP2 knockdown significantly reduced tumor growth and enhanced apoptosis in a xenograft model. These findings support that MYCBP2 promotes AML progression at least in part by negatively regulating KIF14 stability, highlighting MYCBP2 as a potential therapeutic target. Future research should explore targeted therapies aimed at MYCBP2 and its downstream pathways to improve treatment strategies for AML.

Acute myeloid leukemia (AML) is a highly heterogeneous hematological malignancy characterized by the clonal proliferation of myeloid progenitor cells (1). Despite advancements in treatment modalities, achieving a complete and lasting remission remains a significant challenge, largely due to the complexity of the disease and its underlying molecular mechanisms. Recent studies have highlighted various genetic and epigenetic alterations that contribute to the pathogenesis of AML, indicating that a deeper understanding of these processes is critical for developing targeted therapies (2, 3). Among the numerous genes implicated in AML, MYCBP2 (MYC binding protein 2) has emerged as a potential player whose role is yet to be fully elucidated (4).

While several oncogenic pathways are known to drive AML progression, the specific contributions of MYCBP2 have not been thoroughly investigated. This gene encodes an E3 ubiquitin ligase, a class of enzymes that mediates the post-translational modification of proteins through ubiquitination, thereby influencing various cellular processes, including cell proliferation, apoptosis, and response to stress (5). In parallel, the ubiquitin–proteasome system (UPS) is increasingly recognized as both a driver and a therapeutic vulnerability in myeloid malignancies, with several E3 ligases shown to modulate leukemic signaling and treatment response; this context provides a rationale to interrogate defined E3–substrate axes in AML (6). Notably, MYCBP2 has been associated with the regulation of key signaling pathways involved in cancer, such as the p38 MAPK pathway, implicating it in the modulation of tumorigenic processes (7, 8). Mechanistically, MYCBP2 is an atypical RING-Cys-Relay E3 that can relay ubiquitin *via* tandem catalytic cysteines and, in some contexts, form ester-linked ubiquitin on Ser/Thr residues, expanding the chemical repertoire by which E3 ligases control substrate stability and function; such properties make MYCBP2 a plausible regulator of mitotic and survival programs in leukemia (9). However, its precise role in AML and the implications for patient prognosis remain largely unexplored.

The current literature indicates a critical gap in understanding how MYCBP2 interacts with other oncogenes, particularly KIF14 (kinesin family member 14), and how these interactions may influence AML pathogenesis (10, 11). KIF14 has been identified as an important regulator in various cancers, and its expression levels have been correlated with patient outcomes in several malignancies (12). Functionally, KIF14 is a mitotic kinesin that coordinates spindle dynamics and cytokinesis; while it is frequently upregulated and linked to adverse outcomes across multiple solid tumors, its lineage-specific roles in hematologic contexts are less well-defined, with emerging datasets suggesting that kinesin pathways—including those involving KIF14—may contribute to AML biology and prognosis (13, 14). Investigating the functional relationship between MYCBP2 and KIF14 could provide valuable insights into the molecular underpinnings of AML and highlight potential therapeutic targets.

To address this knowledge gap, our study employs a multifaceted approach that integrates bioinformatics analysis, *in vitro* experiments, and *in vivo* models to elucidate the regulatory role of MYCBP2 in AML. Bioinformatics analysis allows for the comprehensive evaluation of gene expression data from multiple databases, while *in vitro* and *in vivo* experiments facilitate the direct validation of findings. This integrated methodology not only enhances the robustness of the results but also enables a more nuanced understanding of the molecular interactions at play in AML (15, 16).

We show that MYCBP2 is upregulated in AML, associates with adverse prognosis, and promotes leukemic cell growth by destabilizing KIF14 *via* the ubiquitin–proteasome pathway. Modulating MYCBP2 alters AML cell proliferation and viability, supporting MYCBP2 as a potential therapeutic target and motivating evaluation in other malignancies (17, 18).

## Results

### The upregulation of MYCBP2 is associated with adverse clinical outcomes in AML

**Figure 1:**
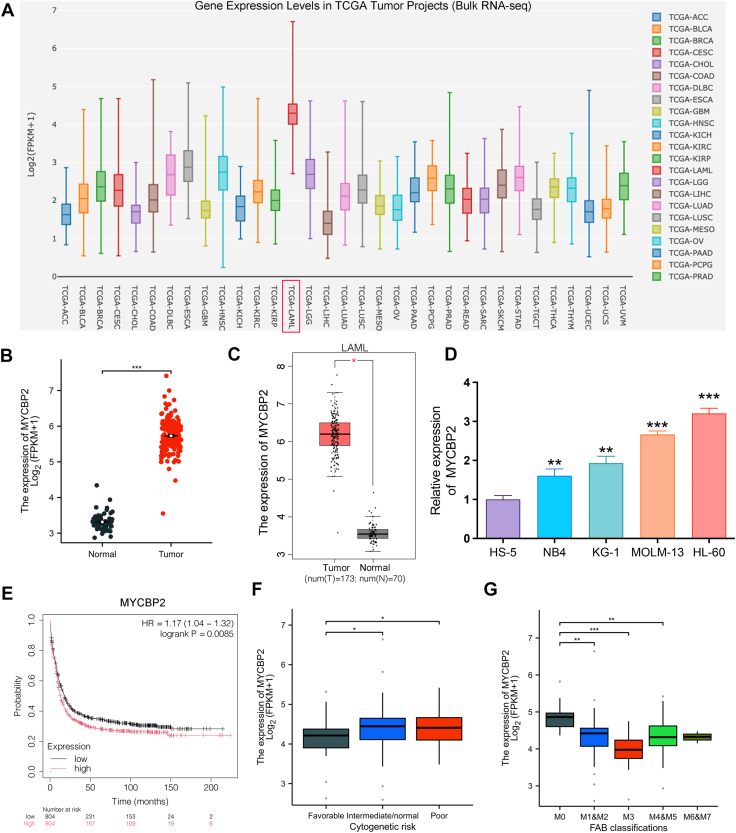
**MYCBP2 is overexpressed in AML and associated with adverse clinical outcomes**. *A*, expression across tumors in the Cancer Single-cell Expression Map; LAML, acute myeloid leukemia (TCGA-LAML). *B*, TCGA data showing MYCBP2 expression in AML *versus* normal tissues (T = 173, N = 70). *C*, GEPIA validation of MYCBP2 upregulation in AML. *D*, MYCBP2 expression in AML cell lines compared to HS-5(n = 3). *E*, Kaplan-Meier survival curves: high MYCBP2 correlates with poor overall survival. *F*, MYCBP2 expression stratified by cytogenetic risk groups. *G*, MYCBP2 expression in FAB subtypes (FAB, French–American–British classification of AML subtypes M0–M7; M0 = minimally differentiated AML; M0 = 19, M1+M2 = 88, M3 = 21, M4+M5 = 64, M6+M7 = 6). mean ± SD; ∗*p*< 0.05, ∗∗*p*< 0.01, ∗∗∗*p*< 0.001.

In a comparative analysis of MYCBP2 expression across various tumors using the Cancer Single-cell Expression Map, we observed that MYCBP2 is abnormally overexpressed in acute myeloid leukemia (LAML; TCGA-LAML) (Fig. 1*A*). Furthermore, an examination of TCGA data revealed that the expression of MYCBP2 in LAML samples is significantly higher than that in normal tissues (Fig. 1*B*). This finding was further validated through the GEPIA database, which corroborated the elevated expression of MYCBP2 in LAML (Fig. 1*C*). Additionally, we found that MYCBP2 expression in multiple AML cell lines surpassed that of HS-5 cells (Fig. 1*D*). To assess the impact of MYCBP2 on the prognosis of AML, we employed Kaplan-Meier survival curves. The results indicated that low expression levels of MYCBP2 are associated with improved overall survival (OS) outcomes (Fig. 1*E*). In the TCGA AML cohort, AML patients classified into the favorable risk group exhibited significantly reduced mRNA levels of MYCBP2. Moreover, high expression of MYCBP2 was significantly correlated with intermediate and high cytogenetic risk groups among AML patients stratified by varying cytogenetic risks (Fig. 1*F*). Notably, Within the French–American–British (FAB) classification, MYCBP2 expression was significantly higher in AML-M0 (19) (minimally differentiated AML) (Fig. 1*G*).

### Knockdown of MYCBP2 inhibits AML cell proliferation and promotes apoptosis

**Figure 2:**
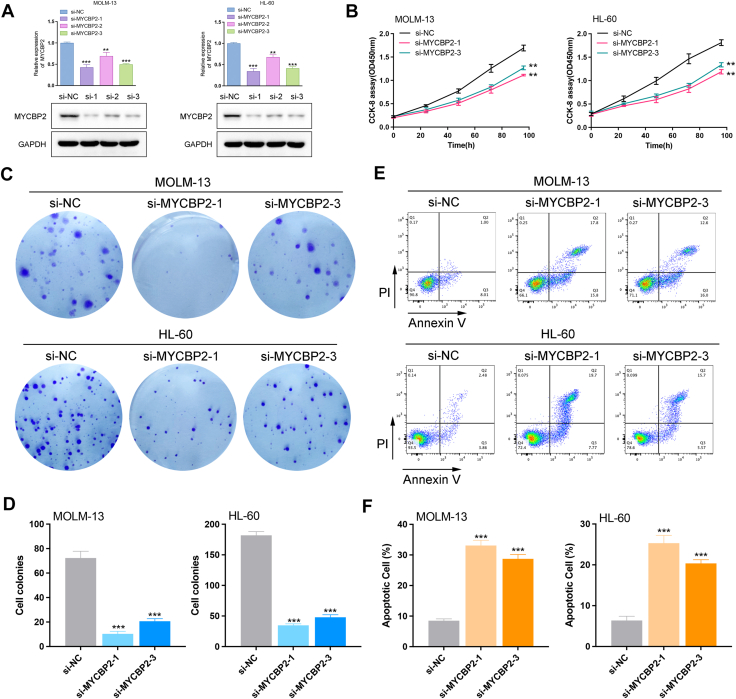
**MYCBP2 knockdown inhibits AML cell proliferation and induces apoptosis**. *A*, qRT-PCR and Western Blot validation of MYCBP2 siRNA knockdown in MOLM-13 and HL-60 cells. *B*, CCK-8 assay: reduced viability post-MYCBP2 knockdown. *C–D*, colony formation assay: suppressed clonogenicity in siRNA-treated cells. *E–F*, flow cytometry: increased apoptosis (Annexin V/PI staining) in MYCBP2-knockdown cells. n = 3, mean ± SD; ∗*p*< 0.05, ∗∗*p*< 0.01, ∗∗∗*p*< 0.001.

To investigate the functional role of MYCBP2 *in vitro*, we utilized siRNA (si-MYCBP2-1, si-MYCBP2-2, si-MYCBP2-3) to knock down MYCBP2 in MOLM-13 and HL-60 cells, followed by qRT-PCR and Western Blot to assess knockdown efficiency (Fig. 2*A*). Subsequently, we evaluated the impact of MYCBP2 knockdown on cell viability using the CCK8 assay. The results demonstrated that knockdown of MYCBP2 significantly inhibited the proliferative capacity of both MOLM-13 and HL-60 cells (Fig. 2*B*). Furthermore, we conducted colony formation assays to further investigate the effect of MYCBP2 on cell proliferation. The findings revealed that the knockdown of MYCBP2 markedly suppressed colony formation in both MOLM-13 and HL-60 cells (Fig. 2, *C* and *D*). Additionally, we employed an Annexin V/PI flow cytometric apoptosis assay to elucidate the specific role of MYCBP2 in apoptosis. Our data indicated that knockdown of MYCBP2 resulted in an increased percentage of apoptosis in MOLM-13 and HL-60 cells (Fig. 2, *E* and *F*).

### Functional analysis of MYCBP2 in AML

**Figure 3:**
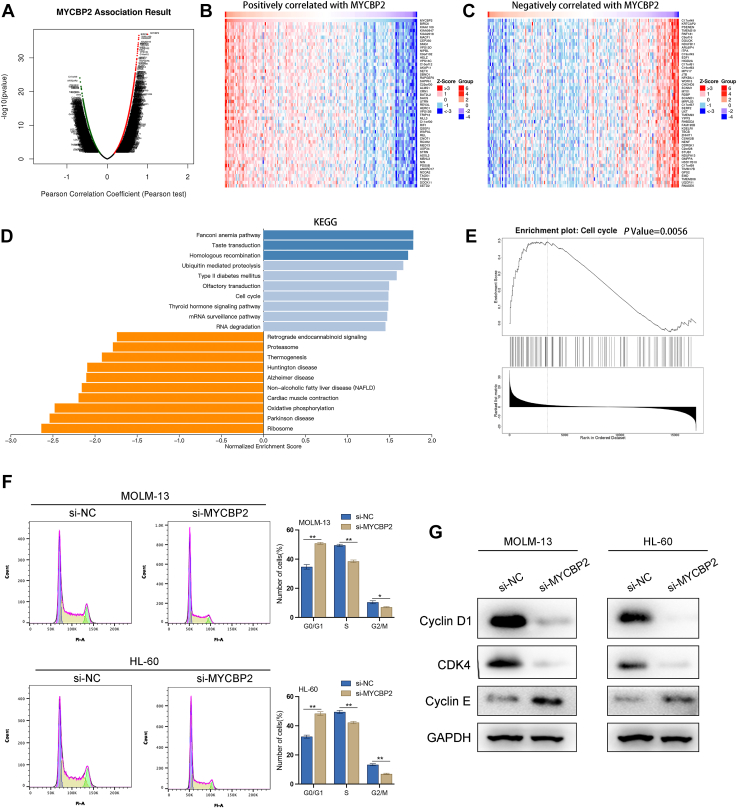
**MYCBP2 regulates cell cycle progression in AML**. *A*, Volcano plot of MYCBP2 co-expressed genes generated using LinkedOmics. *B–C*, Heatmap showing the top 50 positively and negatively correlated genes with MYCBP2. *D*, KEGG pathway enrichment analysis of MYCBP2 co-expressed genes. *E*, gene Set Enrichment Analysis (GSEA) linking MYCBP2 to cell cycle regulation. *F*, flow cytometry analysis of G0/G1 phase arrest in MYCBP2-knockdown cells. *G*, Western blot analysis showing downregulation of Cyclin D1 and CDK4, and upregulation of Cyclin E1. n = 3, mean ± SD; ∗*p*< 0.05, ∗∗*p*< 0.01, ∗∗∗*p*< 0.001.

To explore the mechanistic role of MYCBP2 in AML, we employed LinkedOmics to analyze genes co-expressed with MYCBP2 in AML. A total of 8109 genes were found to be significantly associated with MYCBP2, of which 4351 genes exhibited a positive correlation and 3758 genes displayed a negative correlation (FDR < 0.05; Figure 3*A*). We visualized the top 50 genes that were significantly positively or negatively associated with MYCBP2 (Fig. 3, *B* and *C*). Furthermore, KEGG enrichment analysis revealed that the genes co-expressed with MYCBP2 in AML were enriched in pathways related to Homologous Recombination, Ubiquitin-Mediated Proteolysis, Cell Cycle, and RNA Degradation (Fig. 3*D*). Additionally, Gene Set Enrichment Analysis (GSEA) indicated a close relationship between high MYCBP2 expression and cell cycle regulation (Fig. 3*E*). Subsequently, we utilized flow cytometry to investigate the specific role of MYCBP2 in the cell cycle. Our data indicated that knockdown of MYCBP2 led to an accumulation of MOLM-13 and HL-60 cells in the G0/G1 phase of the cell cycle (Fig. 3*F*). To further validate these findings, we conducted Western blot analysis to assess the expression levels of cell cycle proteins and cyclin-dependent kinases. The results demonstrated that in MYCBP2-knockdown AML cells, the expression of Cyclin D1 and CDK4 was downregulated, while Cyclin E1 expression was elevated (Fig. 3*G*). In conclusion, these findings suggest that MYCBP2 plays a critical role in regulating the cell cycle in AML, potentially contributing to the proliferation and survival of AML cells. This highlights MYCBP2 as a promising therapeutic target for intervention in AML treatment strategies.

### MYCBP2 regulates the ubiquitination process of KIF14

**Figure 4:**
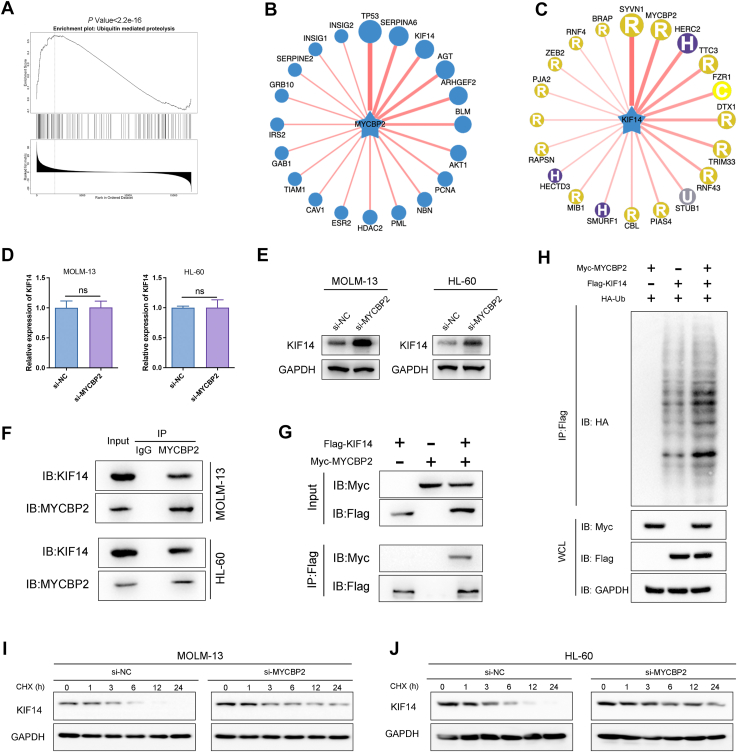
**MYCBP2 destabilizes KIF14 *via* ubiquitin-proteasome system**. *A*, GSEA associating MYCBP2 with ubiquitin-mediated proteolysis. *B–C*, UbiBrowser prediction of MYCBP2-KIF14 interaction (confidence score: 0.746). *D*, RT-qPCR confirms no significant change in KIF14 mRNA expression after MYCBP2 knockdown (n.s., *p*> 0.05). *E*, Western blot analysis shows increased KIF14 protein levels in MYCBP2-knockdown MOLM-13 and HL-60 cells (∗*p*< 0.05 vs. si-NC). *F*, Co-IP confirming endogenous MYCBP2-KIF14 interaction. *G*, exogenous co-IP in 293T cells (Myc-MYCBP2/Flag-KIF14). *H*, ubiquitination assay demonstrating that MYCBP2 enhances the ubiquitination of KIF14. *I–J*, Cycloheximide (CHX) chase experiment showing prolonged half-life of KIF14 in MYCBP2-knockdown cells. n = 3, mean ± SD; ∗*p*< 0.05, ∗∗*p*< 0.01, ∗∗∗*p*< 0.001.

**Fig S1:**
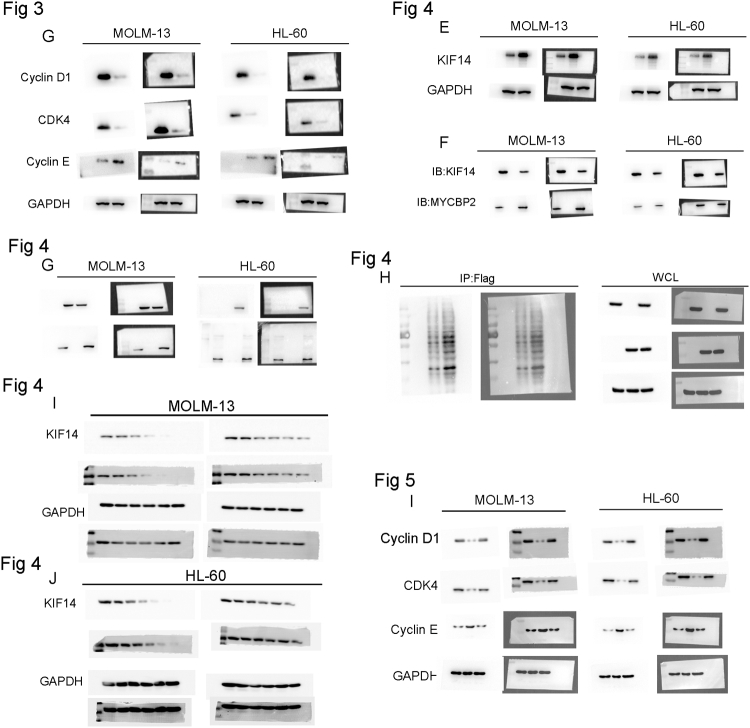


Based on the analysis of co-expressed genes with MYCBP2 in AML using the Linked Omics database, Gene Set Enrichment Analysis (GSEA) indicated that high expression levels of MYCBP2 are closely associated with Ubiquitin-Mediated Proteolysis (Fig. 4*A*). Consequently, we utilized the UbiBrowser database to identify potential substrates regulated by MYCBP2 through ubiquitination. The top three candidates identified were tumor protein p53 (TP53), serpin family A member 6 (SERPINA6; corticosteroid-binding globulin, CBG), and kinesin family member 14 (KIF14). Further substrate prediction using the UbiBrowser database revealed that only KIF14 exhibited a high confidence score of 0.746 with MYCBP2 (Fig. 4, *B* and *C*), suggesting that KIF14 is a potential substrate of MYCBP2. Our experimental data demonstrated that knockdown of MYCBP2 expression in AML cells did not affect the mRNA levels of KIF14, but it resulted in a significant increase in KIF14 protein levels (Fig. 4, *D* and *E*), indicating a post-transcriptional regulatory mechanism. To further explore the interaction between MYCBP2 and KIF14, we performed immunoprecipitation (IP) experiments using an anti-MYCBP2 antibody in AML cells, which confirmed their endogenous interaction (Fig. 4*F*). Additionally, co-immunoprecipitation (co-IP) assays conducted in 293T cells co-transfected with Myc-MYCBP2 and Flag-KIF14 plasmids further validated their interaction (Fig. 4*G*). Subsequently, we co-transfected 293T cells with Myc-MYCBP2, Flag-KIF14, and HA-Ub plasmids, and the results indicated that overexpression of MYCBP2 significantly induced the ubiquitination of KIF14 (Fig. 4*H*). We examined whether KIF14 is regulated by MYCBP2 through the ubiquitin–proteasome pathway. MG132 treatment increased endogenous KIF14 levels and attenuated the difference between control and MYCBP2-silenced cells, supporting that MYCBP2-mediated KIF14 downregulation is proteasome dependent (Fig. S1*A*). We next assessed endogenous KIF14 ubiquitination by immunoprecipitation followed by ubiquitin immunoblotting. MYCBP2 knockdown reduced the ubiquitination level of endogenous KIF14, indicating that MYCBP2 promotes endogenous KIF14 ubiquitination in AML cells (Fig. S1*B*). We further tested the ubiquitin linkage type using linkage-restricted ubiquitin constructs. MYCBP2 preferentially enhanced K48-linked rather than K63-linked ubiquitination of KIF14, and the K48R mutant attenuated MYCBP2-induced ubiquitination. These data indicate that MYCBP2 promotes K48-linked polyubiquitination and proteasomal degradation of KIF14 (Fig. S1*C*). To elucidate the regulatory mechanism by which MYCBP2 affects KIF14 stability, we treated cells with cycloheximide (CHX) to block protein translation. Western blot analysis revealed that the half-life of KIF14 was significantly prolonged in MYCBP2 knockdown cells (Fig. 4, *I* and *J*). We next examined whether MYCBP2 catalytic activity is required for KIF14 regulation. In MYCBP2-depleted cells, re-expression of wild type, but not E3-defective, MYCBP2 restored endogenous KIF14 ubiquitination (Fig. S1*D*). Wild-type MYCBP2 also rescued the reduction in cell viability caused by MYCBP2 knockdown, whereas the E3-defective mutant did not (Fig. S1*E*). Consistently, CHX-chase analysis showed that wild-type MYCBP2 accelerated KIF14 degradation, while the mutant largely lost this effect (Fig. S1*F*). These findings indicate that MYCBP2 E3 ligase activity is required for KIF14 ubiquitination, turnover, and MYCBP2-dependent proliferative phenotypes. In conclusion, these findings suggest that MYCBP2 regulates the ubiquitination and stability of KIF14, thereby influencing its protein levels in AML cells. This regulatory mechanism highlights the potential role of MYCBP2 in modulating the functional dynamics of KIF14, which may contribute to the progression of AML.

### KIF14 contributes to MYCBP2-dependent AML cell phenotypes

**Figure 5:**
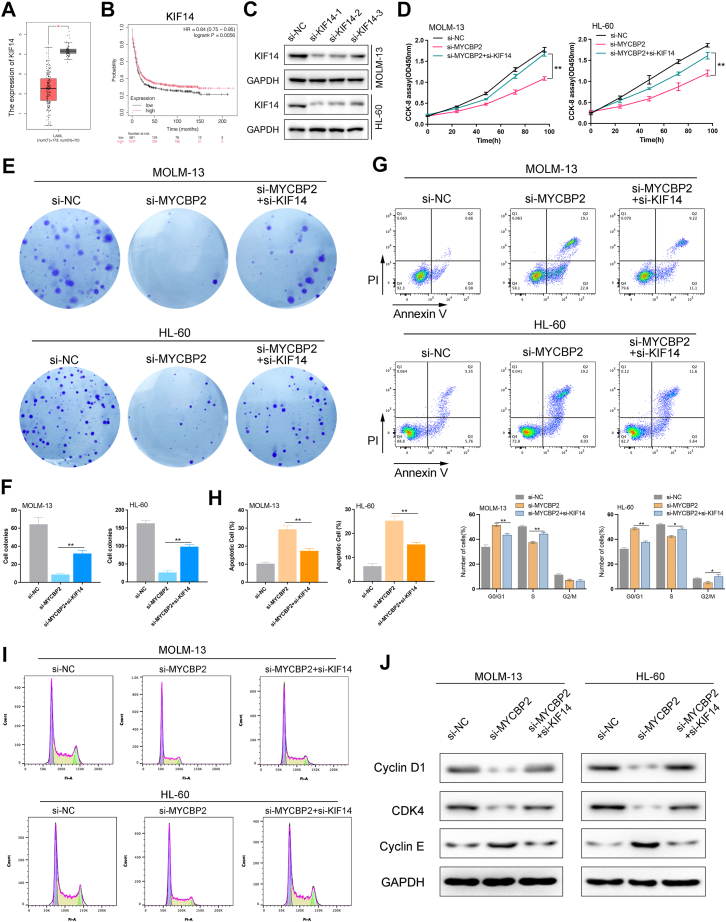
**MYCBP2 promotes AML progression by suppressing KIF14**. *A*, downregulation of KIF14 in acute myeloid leukemia (AML) as analyzed using GEPIA. *B*, Kaplan-Meier survival analysis indicating that high KIF14 expression predicts better overall survival (OS). *C*, Western Blot validation of KIF14 siRNA knockdown in MOLM-13 and HL-60 cells. *D*, CCK-8 assay demonstrating that si-KIF14 partially rescues the viability loss induced by MYCBP2 knockdown. *E–F*, colony formation assay confirming that si-KIF14 reverses the effects of MYCBP2 knockdown. *G–H*, analysis of apoptosis and cell cycle rescue following KIF14 knockdown. *I*, Western blot analysis showing restored levels of Cyclin D1, CDK4, and Cyclin E1. n = 3, mean ± SD; ∗*p*< 0.05, ∗∗*p*< 0.01, ∗∗∗*p*< 0.001.

**Fig S2:**
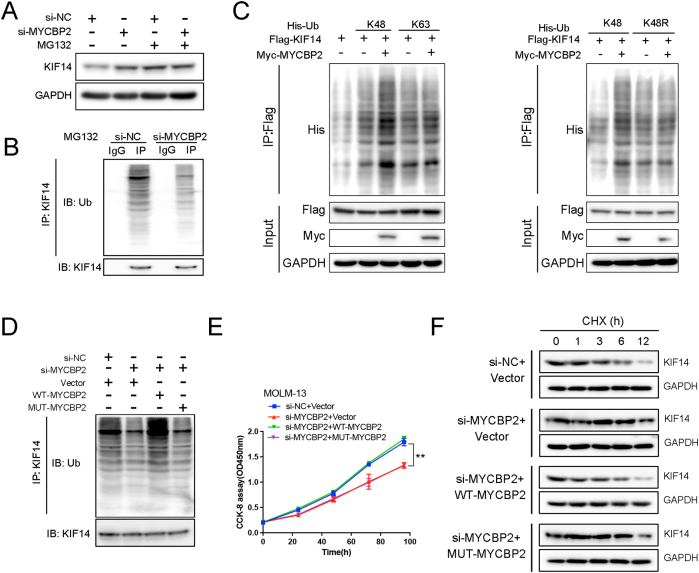


Utilizing the GEPIA database, we discovered that KIF14 is significantly downregulated in LAML (Fig. 5*A*). To evaluate the impact of KIF14 on AML prognosis, we employed Kaplan-Meier survival curves, which revealed that high expression levels of KIF14 are associated with better overall survival (OS) outcomes (Fig. 5*B*). Western blot analysis showed that KIF14 protein expression was markedly reduced after siRNA transfection in MOLM-13 and HL-60 cells (Fig. 5*C*). We next assessed the functional role of KIF14 in AML cells. KIF14 knockdown reduced cell viability in MOLM-13 cells (Fig. S2*A*). It also decreased Bcl-2 and increased cleaved caspase-3, indicating enhanced apoptosis (Fig. S2*B*). In parallel, Cyclin D1 and Cyclin E were reduced after KIF14 silencing, supporting impaired cell-cycle progression (Fig. S2*C*). To validate the hypothesis that MYCBP2 facilitates the malignant progression of AML cells through the ubiquitin-mediated degradation of KIF14, we conducted co-transfection rescue experiments using si-MYCBP2 and si-KIF14. The results from the CCK8 assay indicated that knockdown of MYCBP2 inhibited the viability of MOLM-13 and HL-60 cells; however, knockdown of KIF14 partially reversed this effect (Fig. 5*D*). Similarly, colony formation assays demonstrated that the knockdown of KIF14 reversed the reduction in colony formation induced by MYCBP2 knockdown (Fig. 5, *E* and *F*). Annexin V/PI flow cytometric apoptosis assay further revealed that KIF14 knockdown counteracted the increase in apoptosis percentage caused by MYCBP2 knockdown (Fig. 5, *G* and *H*). Additionally, cell cycle analysis indicated that KIF14 knockdown reversed the G0/G1 phase arrest induced by MYCBP2 knockdown (Fig. 5*I*). Western blot analysis confirmed that the knockdown of KIF14 partially reversed the downregulation of Cyclin D1 and CDK4, as well as the upregulation of Cyclin E1 caused by MYCBP2 knockdown (Fig. 5*J*). We then examined whether KIF14 participates in MYCBP2-mediated phenotypes. MYCBP2 overexpression increased cell viability, whereas co-expression of KIF14 attenuated this effect (Fig. S2*D*). A similar pattern was observed for apoptosis-related proteins. MYCBP2 overexpression increased Bcl-2 and reduced cleaved caspase-3, while KIF14 re-expression partially reversed these changes (Fig. S2*E*). Cyclin D1 and Cyclin E were also upregulated by MYCBP2 overexpression and diminished after KIF14 re-expression (Fig. S2*F*). In conclusion, these findings suggest that MYCBP2 exerts its oncogenic effects in AML, at least in part, by negatively regulating KIF14. This regulatory mechanism highlights the potential of targeting MYCBP2 and its downstream pathways as a therapeutic strategy for AML treatment.

### Knockdown of MYCBP2 inhibits in vivo progression of AML

**Figure 6:**
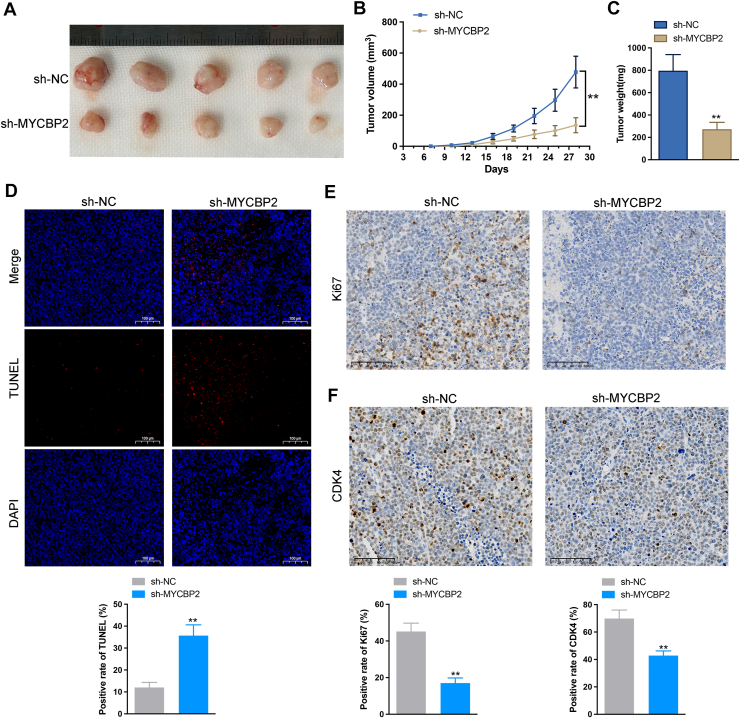
**MYCBP2 knockdown suppresses AML tumor growth *in vivo*.***A*, tumor growth curves in xenograft mice comparing the sh-MYCBP2 group to the sh-NC group. *B*, representative images of tumors. *C*, Reduced tumor weight observed in the sh-MYCBP2 group. *D*, TUNEL staining indicating increased TUNEL-positive nuclei in tumors from the sh-MYCBP2 group. (*E–F*) Immunohistochemistry (IHC) analysis showing decreased expression of Ki67 and CDK4. n = 5, mean ± SD; ∗*p*< 0.05, ∗∗*p*< 0.01, ∗∗∗*p*< 0.001.

To further investigate the impact of MYCBP2 on tumor growth *in vivo*, we injected HL-60 stable cell lines with sh-NC and sh-MYCBP2 into BALB/c nude mice to establish a xenograft tumor model. The results from the xenograft tumor model demonstrated that knockdown of MYCBP2 significantly reduced the rate of tumor growth (Fig. 6, *A* and *B*) and resulted in a decrease in tumor weight (Fig. 6*C*). Moreover, TUNEL fluorescence staining revealed that MYCBP2 knockdown markedly increased DNA fragmentation (TUNEL-positive nuclei) in tumor tissues (Fig. 6*D*). Immunohistochemical analysis further indicated that the proliferation marker KI67 was decreased following MYCBP2 knockdown (Fig. 6*E*), and the expression of the cell cycle protein CDK4 was also significantly reduced (Fig. 6*F*). In conclusion, these findings suggest that MYCBP2 plays a critical role in promoting tumor growth in AML. The inhibition of MYCBP2 not only suppresses tumor proliferation but also enhances TUNEL-positive nuclei, highlighting its potential as a therapeutic target for the treatment of AML.

## Discussion

Acute myeloid leukemia (AML) is a complex and heterogeneous hematological malignancy characterized by the clonal proliferation of myeloid precursors in the bone marrow and peripheral blood (20, 21). The disease presents significant clinical challenges due to its diverse genetic abnormalities and variable response to treatment. Despite advancements in understanding the molecular underpinnings of AML, the prognosis remains poor for many patients, particularly those with high-risk cytogenetic profiles or specific molecular mutations (22, 23). The identification of novel therapeutic targets is crucial for improving treatment outcomes, as current therapies, including intensive chemotherapy and stem cell transplantation, are often insufficient in achieving durable remissions in a substantial proportion of patients (24, 25).

This study investigates the role of MYCBP2, a putative E3 ubiquitin ligase, in the pathogenesis of AML and its potential as a therapeutic target. Preliminary findings indicate that MYCBP2 is overexpressed in AML samples compared to normal tissues, and its high expression correlates with adverse clinical outcomes. The study aims to elucidate the functional dynamics of MYCBP2, particularly its interaction with KIF14, and to explore the implications of these interactions for AML treatment strategies. By employing a combination of bioinformatics analyses and experimental validation, this research seeks to fill the existing gaps in understanding the role of MYCBP2 in AML progression and to highlight its potential as a novel therapeutic target (26). In line with this objective, emerging evidence indicates that the ubiquitin–proteasome system and specific E3 ligases can shape leukemic signaling and therapeutic response, providing a rationale to interrogate defined E3–substrate axes in AML (6, 27).

The elucidation of MYCBP2's involvement in AML unveils critical insights into the molecular mechanisms underpinning this malignancy. Our study indicates that MYCBP2 is overexpressed in AML tissues, correlating with poor clinical outcomes and suggesting its role as an oncogenic driver in the pathogenesis of AML. The identification of MYCBP2 as a negative regulator of KIF14 through ubiquitin-mediated degradation sheds light on its influence on cell proliferation and apoptosis, vital processes in cancer progression. This interaction highlights the significance of MYCBP2 in modulating the ubiquitin-proteasome system, thereby impacting the stability of KIF14, which may act as a tumor-suppressive factor in AML. Collectively, KIF14 is an important, but likely not exclusive, mediator of MYCBP2’s effects; additional substrates/pathways may cooperate and warrant further investigation. These findings align with previous studies that emphasize the importance of the ubiquitin-proteasome pathway in cancer biology, particularly in the context of targeting E3 ligases like MYCBP2 for therapeutic interventions (4, 28). By delineating these molecular pathways, our research provides a foundation for developing novel therapeutic strategies aimed at enhancing patient outcomes in AML. Consistent with this view, our data position the MYCBP2–KIF14 ubiquitination axis as a regulator of leukemic growth and a potentially actionable node in AML proteostasis, in agreement with recent reports implicating E3–substrate pairs in treatment response (29).

In addition to its molecular implications, our findings significantly enhance the understanding of gene function and cellular behavior in AML. The knockdown of MYCBP2 resulted in reduced cell proliferation and increased apoptosis in AML cell lines, indicating that MYCBP2 plays a pivotal role in the survival of these malignant cells. Furthermore, flow cytometry analyses revealed that MYCBP2 knockdown led to an accumulation of cells in the G0/G1 phase of the cell cycle, effectively halting their progression. This blockade of cell cycle dynamics underscores the adaptability of AML cells to therapeutic pressures and suggests that targeting MYCBP2 may disrupt their survival mechanisms. Our results resonate with previous research demonstrating the critical roles of cell cycle regulators and apoptotic pathways in cancer proliferation (16, 30). Such insights into the functional dynamics of MYCBP2 and KIF14 contribute to a deeper understanding of AML's complexity and the potential for targeted therapies. Notably, although KIF14 drives proliferation in many solid tumors, lineage-specific roles are plausible in hematologic disease; our findings suggest that modulating KIF14 stability can influence AML cell-cycle behavior and support future studies to define ubiquitin linkage types, map degron determinants, and evaluate pharmacologic perturbations of the UPS for therapeutic tractability (14).

Moreover, the implications of MYCBP2's regulatory role extend into immune mechanisms, particularly in the context of treatment responses. Given the relationship between MYCBP2 expression and immune cell infiltration in the tumor microenvironment, these findings may influence the effectiveness of immunotherapeutic strategies in AML. The correlation of MYCBP2 with inflammatory responses suggests that it could be a critical player in modulating the tumor's immune landscape, thereby affecting patient outcomes (4, 31). As the field moves toward integrating immunotherapy with existing treatment modalities, understanding how MYCBP2 interacts with immune pathways may provide valuable insights for enhancing therapeutic efficacy and overcoming resistance in AML. This research not only elucidates the biological underpinnings of AML progression but also opens new avenues for targeted and combination therapies that could significantly improve clinical outcomes.

This study is subject to several limitations that warrant consideration. Primarily, the lack of *in vivo* clinical validation may restrict the applicability of our findings to broader patient populations. While our analysis utilized multiple datasets to enhance the robustness of our results, the inherent variability across these databases could introduce biases that affect the generalizability of the conclusions drawn. Additionally, the relatively small sample size in some experimental studies may limit the statistical power necessary for definitive claims regarding MYCBP2's role in AML. Future investigations should aim to include larger cohorts and clinical samples to reinforce the clinical relevance of MYCBP2 as a prognostic biomarker and therapeutic target in AML.

In summary, our research identifies MYCBP2 as a pivotal regulator of AML progression, primarily through its interaction with KIF14, affecting cell proliferation and apoptosis. The findings underscore MYCBP2's potential as a novel therapeutic target, suggesting that interventions aimed at modulating its expression or activity may enhance treatment strategies for AML. Furthermore, this study opens avenues for future research to explore the broader implications of MYCBP2 in other malignancies, thereby contributing to our understanding of cancer biology and therapeutic development.

## Experimental procedure

### Bioinformatics analysis

Bioinformatics analysis was conducted using various online databases to evaluate the expression of MYCBP2 and KIF14 in AML samples. The TCGA database was utilized to obtain expression data for MYCBP2 and KIF14 in LAML samples (https://www.cancer.gov/tcga). The GEPIA database was accessed to retrieve expression profiles of MYCBP2 and KIF14 in AML patients compared to normal controls (http://gepia.cancer-pku.cn/). Kaplan–Meier analyses were performed using KMplot (https://kmplot.com/analysis/) on available AML cohorts to evaluate the prognostic impact of MYCBP2 and KIF14. Additionally, the expression of MYCBP2 across different tumor types was analyzed using the Cancer Single-cell Expression Map (CancerSCEM) (http://cancerscem.org/). LinkedOmics was employed to identify genes co-expressed with MYCBP2, yielding a list of significantly associated genes along with KEGG pathway enrichment and Gene Set Enrichment Analysis (GSEA) (http://www.linkedomics.org/). A false discovery rate (FDR) of less than 0.05 was set as the significance threshold, and the top 50 positively and negatively correlated genes were visualized. Furthermore, the UbiBrowser database was utilized to identify potential downstream substrates of MYCBP2 and explore its association with KIF14-related ubiquitin E3 ligases (http://ubibrowser.org/).

### Cell culture and transfection

MOLM-13 and HL-60 cells (purchased from Wuhan Procell Life Science & Technology Co., Ltd) were cultured in RPMI-1640 medium supplemented with 10% fetal bovine serum (FBS) and 1% penicillin-streptomycin, maintained at 37 °C in a 5% CO_2_ incubator until reaching 70% to 80 confluence. For transfection, Lipofectamine 2000 was employed as the transfection reagent. An appropriate amount of Lipofectamine 2000 was added to a sterile centrifuge tube, followed by the addition of si-NC, si-MYCBP2, and si-KIF14, mixed gently and allowed to stand for 15 min to form transfection complexes. Subsequently, cells were collected and washed once with PBS to remove FBS, then resuspended in serum-free RPMI-1640 medium at a concentration of 1 × 10^6^cells/ml. Transfection conditions were optimized by adjusting cell density, reagent dosage, and nucleic acid input before formal experiments. The transfection complexes were slowly added to the cell suspension, mixed gently, and distributed into 24-well plates, with 1 ml of cell suspension added to each well, ensuring approximately 2.5 × 10^5^cells per well. RT–qPCR was performed at 48 h post-transfection to measure mRNA levels; at 72 h, Western blotting was used to assess target-protein levels.

### RT-qPCR

To assess the expression levels of MYCBP2 and KIF14, total RNA was extracted from AML cells and tumor tissues using TRIzol reagent. The concentration and purity of the extracted RNA were determined using a NanoDrop spectrophotometer. cDNA was synthesized using a reverse transcription kit, with the reverse transcription reaction carried out at 37 °C for 1 h. Subsequently, real-time quantitative PCR (RT-qPCR) analysis was performed using TB Green Premix Ex Taq, following the PCR conditions: initial denaturation at 95 °C for 30 s, followed by 40 cycles of denaturation at 95 °C for 5 s, annealing at 60 °C for 30 s, and extension at 72 °C for 30 s. The primer sequences used were as follows: MYCBP2: Forward: 5′-GGATGATACCACCAGGAACTCAG-3′,

Reverse: 5′- GCCTATCTGCTCACTCTGAAGG -3′

KIF14: Forward: 5′- GCACTTTCGGAACAAGCAAACCA -3′,

Reverse: 5′- ATGTTGCTGGCAGCGGGACTAA -3′

GAPDH: Forward: 5′- GTCTCCTCTGACTTCAACAGCG -3′,

Reverse: 5′- ACCACCCTGTTGCTGTAGCCAA -3′

Relative expression levels were calculated using the 2^-ΔΔCt^ method, with GAPDH serving as the internal control.

### Cell counting Kit-8 (CCK-8) assay

To evaluate the proliferative capacity of AML cells, the CCK-8 assay was performed. Transfected MOLM-13 and HL-60 cells were seeded in 96-well plates at a density of 1 × 10^4^cells per well. At 24, 48, 72, and 96 h post-seeding, 10 μl of CCK-8 reagent was added to each well, and the cells were incubated for an additional 2 h. The optical density (OD) was measured at 450 nm using a microplate reader.

### Colony formation assay

Transfected MOLM-13 and HL-60 cells were seeded at a density of 500 cells per well in 6-well plates coated with agarose and cultured for 14 days to allow colony formation. The medium was changed every 3 to 4 days during the culture period. After 14 days, cells were fixed with 4% paraformaldehyde for 30 min, followed by staining with Crystal violet for 10 min. Excess dye was washed away with water, and the number of colonies in each well was counted, with more than 50 colonies considered positive.

### Western Blot

Total protein was extracted from cells in each treatment group, and protein concentration was determined using a BCA protein assay kit. Equal amounts of protein samples were separated by SDS-PAGE and transferred to PVDF membranes. The membranes were blocked with 5% non-fat milk for 1 h, followed by overnight incubation at 4 °C with specific primary antibodies against MYCBP2, KIF14, Cyclin D1, CDK4, Cyclin E1, HA, and GAPDH (1:1000). After washing, HRP-conjugated secondary antibodies (1:5000) were added, and the membranes were developed using ECL reagents. Protein bands were visualized and quantified using an imaging system.

### Flow cytometry analysis

For cell cycle analysis, transfected AML cells were collected and fixed with 70% cold ethanol, followed by staining with propidium iodide (PI) according to the manufacturer's instructions. Flow cytometry was performed to analyze the distribution of cells in the G0/G1, S, and G2/M phases. For cell apoptosis analysis, cells were treated with an Annexin V-FITC/PI apoptosis detection kit, and flow cytometry was used to determine the apoptotic rate, analyzing both early and late apoptotic cell populations.

### Co-immunoprecipitation (Co-IP)

293T cells were transfected (Lipofectamine 2000) with Myc-MYCBP2 and Flag-KIF14 plasmids (and HA-ubiquitin, where indicated) at ∼80% confluence in 6-well plates. To preserve ubiquitin conjugates, cells were treated with MG132 (10 μM, 4–6 h) prior to lysis. Cells were lysed on ice in non-denaturing buffer (50 mM Tris-HCl pH 7.5, 150 mM NaCl, 1% NP-40, 10% glycerol, 1 mM EDTA) supplemented with protease/phosphatase inhibitors and N-ethylmaleimide (NEM, 10 mM). Lysates were clarified by centrifugation and pre-cleared with control agarose for 30 min at 4 °C, then incubated with anti-Flag M2 agarose (or anti-Myc/anti-MYCBP2 with Protein A/G agarose, as indicated) for 2 h at 4 °C. Beads were washed 4 to 5 times with stringent wash buffer (50 mM Tris-HCl pH 7.5, 300 mM NaCl, 0.5% NP-40, 0.1% SDS, 10 mM NEM) and once with detergent-free buffer, and eluted in 2 × Laemmli at 95 °C for 5 min. Immunoblots were probed with anti-HA to detect ubiquitin conjugates and with anti-Flag/anti-Myc to verify bait/prey. Inputs (2–5%), beads-only/IgG controls, and whole-cell lysates were run in parallel with matched exposures.

### TUNEL assay

The TUNEL assay was performed on tumor tissue sections to detect apoptosis-associated DNA fragmentation. Tumor tissue sections were fixed in 4% paraformaldehyde and dehydrated using gradient ethanol. Following the manufacturer's instructions, TUNEL reaction solution was applied to the sections for staining, and DAPI was used to stain the nuclei. TUNEL-positive cells were observed using a fluorescence microscope, and the proportion of positive cells was calculated.

### Immunohistochemistry (IHC)

Immunohistochemical analysis was conducted on tumor tissue sections to evaluate the expression of Ki67 and CDK4. Tissue sections were fixed in 4% paraformaldehyde, dehydrated using gradient ethanol, and subjected to antigen retrieval. The sections were blocked with 5% bovine serum albumin (BSA) for 1 h, followed by overnight incubation with Ki67 and CDK4 antibodies (1:100). After washing, HRP-conjugated secondary antibodies were added for DAB staining. The sections were observed under a microscope, and the expression levels of CDK4 in tumor tissues were recorded.

### Animal studies and ethics

*In vivo* experiments were conducted using 6- to 8-week-old male BALB/c nude mice. All mice were housed in a specific pathogen-free environment, with sterile feed and water provided. Prior to the experiments, all protocols were approved by the animal ethics committee. To establish a subcutaneous tumor model, HL-60 cells (stably expressing sh-NC or sh-MYCBP2) were resuspended in sterile saline at a concentration of 1 × 10^7^cells/ml and injected subcutaneously into the right flank of the mice with 100 μl of the cell suspension. Mice were monitored regularly for tumor growth, and tumor length and width were measured using calipers to calculate tumor volume (volume = length × width^2^/2). At the end of the experiment, mice were euthanized, and tumor tissues were collected for further analysis.

### Statistical analysis

All experiments were performed with at least three independent biological replicates. For assays involving repeated measurements within the same experiment, technical replicates were included as appropriate, and the averaged values were used for statistical analysis. Data are presented as mean ± standard deviation (SD). Statistical analyses were conducted using GraphPad Prism 9.0. Comparisons between two groups were performed using an unpaired two-tailed Student’s *t* test. Comparisons among three or more groups were analyzed using one-way analysis of variance (ANOVA) followed by the appropriate *post hoc* multiple-comparison test. For time-course experiments involving multiple groups, two-way ANOVA was used when appropriate. Statistical significance was indicated as follows: ∗*p*< 0.05, ∗∗*p*< 0.01 and ∗∗∗*p*< 0.001. A *p* value < 0.05 was considered statistically significant.

## Ethics approval

This study was approved by the Animal Ethics Committee of Zhejiang Chinese Medical University (No. 20230267).

## Data availability

The datasets used and/or analyzed during the current study are available from the corresponding author upon reasonable request.

## Supporting information

This article contains supporting information.

## Conflict of interest

The authors declare that they do not have any conflicts of interest with the content of this article.

## Figures and Tables

**Fig S3:**