# Supplementary material for: Ubiquitin E3 ligase MYCBP2 targets KIF14 and contributes to acute myeloid leukemia progression
Source: J Biol Chem. 2026 Apr 24;302(6):112204. doi: 10.1016/j.jbc.2026.112204 (PMC13202567; doi:10.1016/j.jbc.2026.112204)
Supplement: Supplementary figures [file mmc1.docx]

**Figure S1. MYCBP2 promotes KIF14 ubiquitination and degradation in an E3 activity-dependent manner.**

(A) Western blot analysis showing that MG132 treatment increases endogenous KIF14 protein levels and attenuates the difference between si-NC and si-MYCBP2 groups, indicating that MYCBP2-mediated KIF14 downregulation is proteasome dependent. (B) Endogenous ubiquitination assay showing that MYCBP2 knockdown reduces the ubiquitination level of KIF14 in AML cells. (C) Ubiquitination assays in 293T cells showing that MYCBP2 preferentially enhances K48-linked rather than K63-linked ubiquitination of KIF14, and that the K48R ubiquitin mutant attenuates MYCBP2-induced KIF14 ubiquitination. (D) Rescue ubiquitination assay showing that re-expression of wild-type MYCBP2, but not E3-defective mutant MYCBP2, restores endogenous KIF14 ubiquitination in MYCBP2-depleted cells. (E) CCK-8 assay showing that wild-type MYCBP2, but not mutant MYCBP2, rescues the reduction in cell viability caused by MYCBP2 knockdown in MOLM-13 cells. (F) Cycloheximide (CHX) chase assay showing that wild-type MYCBP2 accelerates KIF14 degradation, whereas mutant MYCBP2 largely loses this effect. n=3, mean ± SD; *P < 0.05, **P < 0.01, ***P < 0.001.

**Figure S2. KIF14 mediates MYCBP2-dependent regulation of AML cell proliferation, apoptosis, and cell-cycle-related proteins.**

(A) CCK-8 assay showing that KIF14 knockdown inhibits the viability of MOLM-13 cells. (B) Western blot analysis showing that KIF14 knockdown decreases Bcl-2 and increases cleaved caspase-3 expression. (C) Western blot analysis showing that KIF14 knockdown reduces Cyclin D1 and Cyclin E expression. (D) CCK-8 assay showing that MYCBP2 overexpression promotes MOLM-13 cell viability, whereas co-expression of KIF14 partially attenuates this effect. (E) Western blot analysis showing that MYCBP2 overexpression increases Bcl-2 and decreases cleaved caspase-3, while KIF14 re-expression partially reverses these changes. (F) Western blot analysis showing that MYCBP2 overexpression upregulates Cyclin D1 and Cyclin E, whereas KIF14 re-expression weakens this effect. n=3, mean ± SD; *P < 0.05, **P < 0.01, ***P < 0.001.
